# SARS-CoV-2 and COVID-19: The most important research questions

**DOI:** 10.1186/s13578-020-00404-4

**Published:** 2020-03-16

**Authors:** Kit-San Yuen, Zi -Wei Ye, Sin-Yee Fung, Chi-Ping Chan, Dong-Yan Jin

**Affiliations:** 1 School of Biomedical Sciences, The University of Hong Kong, 3/F Laboratory Block, 21 Sassoon Road, Pokfulam, Hong Kong; 2 Department of Microbiology, The University of Hong Kong, Pokfulam, Hong Kong

**Keywords:** SARS-CoV-2, COVID-19, 2019 novel coronavirus (2019-nCoV), Novel coronavirus pneumonia (NCP)

## Abstract

Coronavirus disease 2019 (COVID-19) caused by severe acute respiratory syndrome coronavirus 2 (SARS-CoV-2) is an ongoing global health emergency. Here we highlight nine most important research questions concerning virus transmission, asymptomatic and presymptomatic virus shedding, diagnosis, treatment, vaccine development, origin of virus and viral pathogenesis.

The 2019-nCoV causes an ongoing outbreak of lower respiratory tract disease called novel coronavirus pneumonia (NCP) by the Chinese government initially. The disease name was subsequently recommended as COVID-19 by the World Health Organization. Meanwhile, 2019-nCoV was renamed SARS-CoV-2 by the International Committee on Taxonomy of Viruses. As of February 24, 2020, more than 80,000 confirmed cases including more than 2,700 deaths have been reported worldwide, affecting at least 37 countries. The WHO has declared this a global health emergency at the end of January 2020. The epicenter of this ongoing outbreak is in the city of Wuhan in Hubei Province of central China and the Huanan seafood wholesale market was thought to be at least one of the places where SARS-CoV-2 from an unknown animal source might have crossed the species barrier to infect humans.

A pioneering study conducted in the city of Shenzhen near Hong Kong by a group of clinicians and scientists from the University of Hong Kong has provided the first concrete evidence for human-to-human transmission of SARS-CoV-2 [1]. This is an excellent example of how a high-quality clinical study can make a major difference in policy setting. Several important clinical features of COVID-19 have also been documented in this study. First, an attack rate of 83% within the family context is alarmingly high, indicating the high transmissibility of SARS-CoV-2. Second, the clinical manifestations of COVID-19 in this family range from mild to moderate, with more systematic symptoms and more severe radiological abnormalities seen in older patients. Generally, COVID-19 appears to be less severe than SARS. Third, an asymptomatic child was found to have ground-glass opacities in his lung and SARS-CoV-2 RNA in his sputum sample. This finding of asymptomatic virus shedding raises the possibility for transmission of SARS-CoV-2 from asymptomatic carriers to others, which is later confirmed by others [2]. Finally, the presentation of diarrhea in two young adults from the same family also suggests the possibility for gastrointestinal involvement in SARS-CoV-2 infection and fecal–oral transmission. The study has set the stage for the control and management of COVID-19 [1]. The work was completed timely and the investigators showed great courage and leadership in a very difficult time when the Chinese authority failed to recognize widespread person-to-person transmission of SARS-CoV-2 before January 20, 2020.

Several interesting papers on SARS-CoV-2 and COVID-19 have been published in the past few weeks to report on the evolutionary reservoir [3], possible intermediate host [4] and genomic sequence [5] of SARS-CoV-2 as well as clinical characteristics of COVID-19 [6, 7]. In view of these findings and the urgent needs in the prevention and control of SARS-CoV-2 and COVID-19, in this commentary we highlight the most important research questions in the field from our personal perspectives.

The first question concerns how SARS-CoV-2 is transmitted currently in the epicenter of Wuhan. In order to minimize the spreading of SARS-CoV-2, China has locked down Wuhan and nearby cities since January 23, 2020. The unprecedented control measures including suspension of all urban transportation have apparently been successful in preventing further spreading of SARS-CoV-2 to other cities. However, the number of confirmed cases in Wuhan continued to rise. It is therefore crucial to determine whether the rise is due to a large number of infected individuals before the lock down and/or failure in the prevention of widespread intra-familial, nosocomial or community transmission. Based on the number of exported cases from Wuhan to cities outside of mainland China, it was predicted that there might be more than 70,000 individuals infected with SARS-CoV-2 on January 25, 2020 in Wuhan [8]. This should be determined experimentally in Wuhan as discussed below and it will reveal whether the real numbers of infected people and asymptomatic carriers are indeed underestimated severely. In addition to viral RNA detection, measurement of IgM and IgG antibodies as well as antigens would be very helpful. Several representative residential areas should be selected for detailed analysis so that a big picture can be deduced. The analysis should include all healthy and diseased individuals within the area with the aim of identifying people who have recovered from an infection or are having an active infection. The ratio of asymptomatic carriers should also be determined. The analysis should also be extended to detect RNA and antigen of influenza viruses. The activity of seasonal flu in Wuhan also reached a peak at the beginning of 2020. It will be of interest to see whether the flu season had ended and how many people having a fever now are actually infected with influenza virus. Precision control measures for SARS-CoV-2 should be tailor-designed for high-risk groups based on the results of this analysis. Differentiating people having a flu and preventing them from infecting with SARS-CoV-2 in a hospital setting might also be critical.

The second question is how transmissible and pathogenic is SARS-CoV-2 in tertiary and quaternary spreading within humans. Continued transmission of SARS-CoV-2 in Wuhan suggests that tertiary and quaternary spreading has occurred. Compared to the primary and secondary spreading during which SARS-CoV-2 was transmitted from animal to human and from human to human, has the transmission rate increased and has the pathogenicity decreased? Alternatively, is the virus less transmissible after several passages in humans? Retrospective analysis of all confirmed cases in Wuhan should be very informative. The answers to the above questions hold the key to the outcome of the outbreak. If the transmission is weakened, the outbreak may ultimately come to an end at which SARS-CoV-2 is eradicated from humans. On the contrary, if effective transmission can be sustained, the chance is increased that SARS-CoV-2 will become another community-acquired human coronavirus just like the other four human coronaviruses (229E, OC43, HKU1 and NL63) causing common cold only. The basic reproductive number (R_0_) of SARS-CoV-2 has been estimated to be 2.68, resulting in an epidemic doubling time of about 6.4 days [8]. Other estimates of R_0_ could go up to 4, higher than that of SARS-CoV, which is lower than 2. Determining the real R_0_ will shed light on whether and to what extent infection control measures are effective.

The third question relates to the importance of asymptomatic and presymptomatic virus shedding in SARS-CoV-2 transmission. Asymptomatic and presymptomatic virus shedding posts a big challenge to infection control [1, 2]. In addition, patients with mild and unspecific symptoms are also difficult to identify and quarantine. Notably, the absence of fever in SARS-CoV-2 infection (12.1%) is more frequent than in SARS-CoV (1%) and Middle East respiratory syndrome coronavirus (MERS-CoV; 2%) infection [6]. In light of this, the effectiveness of using fever detection as the surveillance method should be reviewed. However, based on previous studies of influenza viruses and community-acquired human coronaviruses, the viral loads in asymptomatic carriers are relatively low [9]. If this is also the case for SARS-CoV-2, the risk should remain low. Studies on the natural history of SARS-CoV-2 infection in humans are urgently needed. Identifying a cohort of asymptomatic carriers in Wuhan and following their viral loads, clinical presentations and antibody titers over a time course will provide clues as to how many of the subjects have symptoms in a later phase, whether virus shedding from the subjects is indeed less robust, and how often they might transmit SARS-CoV-2 to others.

The fourth question relates to the importance of fecal–oral route in SARS-CoV-2 transmission. In addition to transmission via droplets and close contact, fecal–oral transmission of SARS-CoV has been shown to be important in certain circumstances. Gastrointestinal involvement of SARS-CoV-2 infection and isolation of SARS-CoV-2 from fecal samples of patients are in support of the importance of fecal–oral route in SARS-CoV-2 transmission. Although diarrhea was rarely seen in studies with large cohorts [6, 7], the possibility of SARS-CoV-2 transmission via sewage, waste, contaminated water, air condition system and aerosols cannot be underestimated, particularly in cases such as the Diamond Princess cruise ship with 3,700 people, among whom at least 742 have been confirmed to be infected with SARS-CoV-2 plausibly as the result of a superspreading event. Further investigations are required to determine the role of fecal–oral transmission in these cases and within the representative residential areas selected for detailed epidemiological studies in Wuhan as discussed earlier.

The fifth question concerns how COVID-19 should be diagnosed and what diagnostic reagents should be made available. RT-PCR-based SARS-CoV-2 RNA detection in respiratory samples provides the only specific diagnostic test at the initial phase of the outbreak. It has played a very critical role in early detection of patients infected with SARS-CoV-2 outside of Wuhan, implicating that widespread infection of the virus had occurred in Wuhan at least as early as the beginning of 2020. This has also pushed the Chinese authority to acknowledge the severity of the situation. Due to difficulties in sampling and other technical issues in this test, at one point in early February clinically diagnosed patients with typical ground glass lung opacities in chest CT were also counted as confirmed cases in order to have the patients identified and quarantined as early as possible. ELISA kits for detection of IgM and IgG antibodies against N and other SARS-CoV-2 proteins have also been available more recently. This has made specific diagnosis of ongoing and past infection possible. Particularly, seroconversion for IgM antibodies normally occurs a few days earlier than that of IgG. ELISA reagents for detection of SARS-CoV-2 antigens such as S and N are still in urgent need, and would provide another test highly complementary to viral RNA detection.

The sixth question concerns how COVID-19 should be treated and what treatment options should be made available. COVID-19 is a self-limiting disease in more than 80% of patients. Severe pneumonia occurred in about 15% of cases as revealed in studies with large cohorts of patients. The gross case fatality is 3.4% worldwide as of February 25, 2020. This rate is 4.4% for patients in Wuhan, 4.0% for patients in Hubei and 0.92% for patients outside of Hubei. The exceedingly high fatality in Wuhan might be explained by the collapse of hospitals, a large number of undiagnosed patients, suboptimal treatment or a combination of these. Up to date, we still do not have any specific anti-SARS-CoV-2 agents but an anti-Ebola drug, remdesivir, may hold the promise. As a nucleotide analog, remdesivir was shown to be effective in preventing MERS-CoV replication in monkeys. Severity of disease, viral replication, and lung damage were reduced when the drug was administered either before or after infection with MERS-CoV [10]. These results provide the basis for a rapid test of the beneficial effects of remdesivir in COVID-19. Other antiviral agents worthy of further clinical investigations include ribavirin, protease inhibitors lopinavir and ritonavir, interferon α2b, interferon β, chloroquine phosphate, and Arbidol. However, we should also bear in mind the side effects of these antiviral agents. For example, type I interferons including interferon α2b and interferon β are well known for their antiviral activity. Their beneficial effects at an early phase of infection are well expected. However, administration at a later stage carries the risk that they might worsen the cytokine storm and exacerbate inflammation. Notably, steroids have been experimentally used widely in the treatment of SARS and are still preferred by some Chinese physicians in the treatment of COVID-19. It is said to be capable of stopping the cytokine storm and preventing lung fibrosis. However, the window in which steroids might be beneficial to patients with COVID-19 is very narrow. In other words, steroids can only be used when SARS-CoV-2 has already been eliminated by human immune response. Otherwise, SARS-CoV-2 replication will be boosted leading to exacerbation of symptoms, substantial virus shedding, as well as increased risk for nosocomial transmission and secondary infection. In this regard, it will be of interest to determine whether the report of fungal infection in the lungs of some patients in Wuhan might be linked to misuse of steroids. Nevertheless, the screening of new pharmaceuticals, small-molecule compounds and other agents that have potent anti-SARS-CoV-2 effects will successfully derive new and better lead compounds and agents that might prove useful in the treatment of COVID-19.

The seventh question is whether inactivated vaccines are a viable option for SARS-CoV-2. The chance that SARS-CoV-2 will become endemic in some areas or even pandemic has increased in view of its high transmissibility, asymptomatic and presymptomatic virus shedding, high number of patients with mild symptoms, as well as the evidence for superspreading events. Thus, vaccine development becomes necessary for prevention and ultimate eradication of SARS-CoV-2. Inactivated vaccines are one major type of conventional vaccines that could be easily produced and quickly developed. In this approach, SARS-CoV-2 virions can be chemically and/or physically inactivated to elicit neutralizing antibodies. In the case of SARS-CoV and MERS-CoV, neutralizing antibodies were successfully and robustly induced by an inactivated vaccine in all types of animal experiments, but there are concerns about antibody-dependent enhancement of viral infection and other safety issues. While inactivated vaccines should still be tested, alternative approaches include live attenuated vaccines, subunit vaccines and vectored vaccines. All of these merit further investigations and tests in animals.

The eighth question relates to the origins of SARS-CoV-2 and COVID-19. To make a long story short, two parental viruses of SARS-CoV-2 have now been identified. The first one is bat coronavirus RaTG13 found in *Rhinolophus affinis* from Yunnan Province and it shares 96.2% overall genome sequence identity with SARS-CoV-2 [3]. However, RaTG13 might not be the immediate ancestor of SARS-CoV-2 because it is not predicted to use the same ACE2 receptor used by SARS-CoV-2 due to sequence divergence in the receptor-binding domain sharing 89% identity in amino acid sequence with that of SARS-CoV-2. The second one is a group of betacoronaviruses found in the endangered species of small mammals known as pangolins [4], which are often consumed as a source of meat in southern China. They share about 90% overall nucleotide sequence identity with SARS-CoV-2 but carries a receptor-binding domain predicted to interact with ACE2 and sharing 97.4% identity in amino acid sequence with that of SARS-CoV-2. They are closely related to both SARS-CoV-2 and RaTG13, but apparently they are unlikely the immediate ancestor of SARS-CoV-2 in view of the sequence divergence over the whole genome. Many hypotheses involving recombination, convergence and adaptation have been put forward to suggest a probable evolutionary pathway for SARS-CoV-2, but none is supported by direct evidence. The jury is still out as to what animals might serve as reservoir and intermediate hosts of SARS-CoV-2. Although Huanan seafood wholesale market was suggested as the original source of SARS-CoV-2 and COVID-19, there is evidence for the involvement of other wild animal markets in Wuhan. In addition, the possibility for a human superspreader in the Huanan market has not been excluded. Further investigations are required to shed light on the origins of SARS-CoV-2 and COVID-19.

The ninth question concerns why SARS-CoV-2 is less pathogenic. If the reduced pathogenicity of SARS-CoV-2 is the result of adaptation to humans, it will be of great importance to identify the molecular basis of this adaptation. The induction of a cytokine storm is the root cause of pathogenic inflammation both in SARS and COVID-19. SARS-CoV is known to be exceedingly potent in the suppression of antiviral immunity and the activation of proinflammatory response. It is therefore intriguing to see how SARS-CoV-2 might be different from SARS-CoV in interferon-antagonizing and inflammasome-activating properties. It is noteworthy that some interferon antagonists and inflammasome activators encoded by SARS-CoV are not conserved in SARS-CoV-2. Particularly, ORF3 and ORF8 in SARS-CoV-2 are highly divergent from ORF3a and ORF8b in SARS-CoV that are known to induce NLRP3 inflammasome activation. ORF3 of SARS-CoV-2 is also significantly different from the interferon antagonist ORF3b of SARS-CoV. Thus, these viral proteins of SARS-CoV and SARS-CoV-2 should be compared for their abilities to modulate antiviral and proinflammatory responses. The hypothesis that SARS-CoV-2 might be less efficient in the suppression of antiviral response and the activation of NLRP3 inflammasome should be tested experimentally.

Much progress has been made in the surveillance and control of infectious diseases in China after the outbreak of SARS-CoV in 2003. Meanwhile, virological research in the country has also been strengthened. The new disease report and surveillance system did function relatively well during the 2009 pandemic of swine flu. New viral pathogens such as avian influenza virus H7N9 and severe-fever-with-thrombocytopenia syndrome bunyavirus have also been discovered in recent years [11, 12], indicating the strength and vigor of Chinese infectious disease surveillance and virological research. However, the ongoing outbreak of SARS-CoV-2 has not only caused significant morbidity and mortality in China, but also revealed major systematic problems in control and prevention of infectious diseases there. Unfortunately, many of the lessons from the 2003 outbreak have not been learned. Importantly, disease control professionals, practicing physicians and scientists are disconnected in the fight against SARS-CoV-2 and COVID-19. In addition, important decisions were not made by experts in the field. Hopefully, these issues will be dealt with swiftly and decisively during and after the outbreak.

Above we have discussed the two possibilities that this outbreak will unfold. If SARS-CoV-2 is not eliminated from humans through quarantine and other measures, it can still be eradicated by vaccination. If it attenuates to become another community-acquired human coronavirus causing mild respiratory tract disease resembling the other four human coronaviruses associated with common cold, it will not be a disaster either. Before SARS-CoV-2 attenuates further to a much less virulent form, early diagnosis and improved treatment of severe cases hold the key to reduce mortality. We should remain vigilant, but there are grounds for guarded optimism. Redoubling our research efforts on SARS-CoV-2 and COVID-19 will solidify the scientific basis on which important decisions are made.

## Data Availability

Not applicable.
